# Pathological Hallmarks, Clinical Parallels, and Value for Drug Testing in Alzheimer's Disease of the APP[V717I] London Transgenic Mouse Model

**DOI:** 10.4061/2010/417314

**Published:** 2010-09-02

**Authors:** An Tanghe, Annelies Termont, Pascal Merchiers, Stephan Schilling, Hans-Ulrich Demuth, Louise Scrocchi, Fred Van Leuven, Gerard Griffioen, Tom Van Dooren

**Affiliations:** ^1^reMYND NV, Gaston Geenslaan 1, 3001 Heverlee-Leuven, Belgium; ^2^Ablynx NV, Technologiepark 214, 9052 Zwijnaarde-Gent, Belgium; ^3^Probiodrug AG, Weinbergweg 22, 06120 Halle-Saale, Germany; ^4^Amorfix Life Sciences Ltd., 3403 American Drive, Mississauga, ON, Canada L4V1T4; ^5^Experimental Genetics Group LEGTEGG, KULeuven, Campus Gasthuisberg ON1, 3000 Leuven, Belgium

## Abstract

The APP[V717I] London (APP-Ld) mouse model recapitulates important pathological and clinical hallmarks of Alzheimer's disease (AD) and is therefore a valuable paradigm for evaluating therapeutic candidates. Historically, both the parenchymal and vascular amyloid deposits, and more recently, truncated and pyroglutamate-modified Abeta_3(pE)-42_ species, are perceived as important hallmarks of AD-pathology. Late stage symptoms are preceded by robust deficits in orientation and memory that correlate in time with Abeta oligomerization and GSK3*β*-mediated phosphorylation of endogenous murine Tau, all markers that have gained considerable interest during the last decade. Clinical parallels with AD patients and the value of the APP-Ld transgenic mouse model for preclinical *in vivo* testing of candidate drugs are discussed.

## 1. Introduction

Notwithstanding the vast amount of resources invested into discovery and development of new targets and treatments, Alzheimer's disease (AD) remains an indication with enormous unmet needs. One complicating factor for successful drug development is the unknown etiology of idiopathic AD. AD entails noxious aggregation of *β*-amyloid (Abeta) and Tau, representing fundamental processes in disease onset and progression [1–3]. The extracellular Abeta plaques and intracellular neurofibrillary tangles (NFTs) represent key pathological hallmarks, but are not necessarily the primary causes of neuronal toxicity. The underlying molecular mechanisms of plaque and tangle formation, and how they interact, remain largely elusive. It is generally accepted that these processes involve formation of misconfomers of the respective proteins with increased propensity to self-polymerize in a stepwise fashion. It is clear that the process of aggregation is toxic and triggers neuronal degeneration. However, no consensus exists as to the exact nature, composition, or conformation of the protein assemblies that trigger neuronal demise. This situation is reflected by the fact that no validated drug targets are known whose modulation provides a robust therapeutic response. However, given the fundamental involvement of Abeta in AD—which is hypothesized as an early instigator of toxic downstream processes in AD—pharmacological intervention of APP pathobiology (processing, clearance, and aggregation) received most interest from the pharma industry up to now.

The identification of the pathogenic variants of hAPP and hPS1 genes running in families with inheritable AD has enabled the generation of transgenic animal models, mainly rodents, for AD (for a recent review, see [4, 5]). In recent years, the value and limitations of AD rodent models, both in terms of providing a better understanding of disease pathogenesis and progression and aiding the development of drugs for treating disease, have been a topic of intense and recurrent debate in the Alzheimer field, as is illustrated by numerous recent reviews and opinion papers [6, 7]. Although none of the current AD rodent models fully recapitulate all aspects of disease, that is, displaying a progressive development of all specific neuropathological and cognitive aspects of AD, some mouse models reproduce or recapitulate at least several of the most important characteristics [8]. 

In this paper, we review pathological read-outs and introduce two new neuropathological markers of the “APP London” (APP-Ld) mouse model, that is, early-onset aggregation of Abeta and subsequent appearance of truncated, pyroglutamate-modified Abeta3–42 (Abeta_3(pE)–42_) amyloid peptide species in the brain. Furthermore, we discuss clinical parallels with the human Alzheimer patient and the value of the model for preclinical *in vivo* testing of candidate Alzheimer drugs.

## 2. Experimental Procedures

### 2.1. Transgenic Mice

Female transgenic mice in mixed FVB/N × C57Bl/6J background expressing heterozygously hAPP[V717I] under control of the neuron-specific murine *thy1 *gene promoter have been used in this study. The construction of the FVB/N background strain has been described earlier [1, 9, 10]. The F1-hybrid strain was a crossing of heterozygous APP[V717I] males in C57Bl/6J background with wild type FVB/N females. In addition, double transgenic mice overexpressing hAPP[V717I] and hPS1[A246E] were generated by crossbreeding the single hAPP[V717I] mutant with homozygous hPS1[A426E] mice [11]. Genotyping by two independent PCR assays per transgene at the age of three weeks and at the onset of the experiments on DNA extracted from tail biopsies confirmed the respective genotypes.

### 2.2. Animal Care and Handling

All treatments were approved by the Local Committee for Animal Use and were performed in accordance to state and federal regulations. During the time of study, mice had access to prefiltered sterile water and standard mouse chow (Ssniff Ms-H, Ssniff Spezialdiäten GmbH, Soest, Germany) ad libitum and were housed under a reversed day-night rhythm in individual ventilated macrolon T2 cages in accordance to local legislation on animal welfare.

### 2.3. Sacrifice, CSF Collection, and Brain Processing

The mice were anaesthetized with a mixture of Anesketin (Ketamin), Rompun (Xylazin 2%), Atropin and saline (1 : 1 : 1 : 1), and perfused transcardiacally with ice-cold saline. Cerebrospinal fluid (CSF) was collected via an incision in the neck muscles between the skull and the first cervical vertebrae. A puncture into the cisterna magna was given with a 26-gauge needle and 10–20 *μ*L of CSF was collected with a fine glass pipette. The brain was excised from the cranium, and hindbrain and forebrain were separated at the coronal plane. The left and right hemispheres were separated. Routinely, one hemisphere was immersion fixed overnight in phosphate-buffered saline (PBS) containing 4% paraformaldehyde for (immuno) histology, and the other hemisphere was snap-frozen in liquid nitrogen and stored at −80°C until further use in biochemical assays.

### 2.4. Abeta40 and Abeta42 in CSF

Human Abeta40 and Abeta42 concentrations in CSF were measured using commercial ELISA kits according to the manufacturers protocol (Human Amyloid 40 or 42 HS ELISA, Millipore).

### 2.5. Abeta Immunohistochemistry

Sagittal free-floating vibratome sections (40 *μ*m) were stored in PBS containing 0,01% (w/v) sodium azide at 4°C until staining. Sections were washed twice in PBS and quenched with 1.5% (v/v) hydrogen peroxide in PBS and methanol (1 : 1) for 15 minutes to remove endogenous peroxidase activity. After washing the sections three times in PBS containing 0,1% Triton X100 (PBST), sections were blocked for 30 minutes in 10% Fetal Calf Serum (FCS) in PBST and incubated overnight with a biotinylated anti-Abeta antibody in blocking buffer (proprietary anti-Abeta Nanobody against N-terminus of Abeta, reMYND/Ablynx). After rinsing, the sections were incubated in 0,01% trypsin in PBS for 15 minutes at room temperature, followed by incubation with avidin-biotin peroxidase complex (Vectastain Elite ABC, Vector Laboratories). The signal was developed with 3,3′ diaminobenzidine tetrahydrochloride tablets (DAB, ICN). Sections were counterstained with Mayers hematoxylin, dehydrated and mounted in Depex (Depex mounting medium, VWR International). Microscopic images were recorded and digitalized with a 3 CCD color video camera and analyzed with dedicated software (Olympus BX41 microscope, Color view II—Olympus camera, Analysis Five—Cell^∧^D software).

### 2.6. Abeta_x–42_ and Abeta_3(pE)–42_ Levels in Brain

Brain tissue was homogenized in TBS (20 mM Tris-HCl pH 7.6, 137 mM NaCl) containing protease inhibitor cocktail (Complete Mini, Roche), followed by sequential extractions with 1% Triton X-100 in TBS (TBS fraction), 2% SDS in water (SDS fraction), and 70% formic acid (FA fraction). The FA fraction was neutralized with 3.5 M Tris and diluted 1 : 20 in EIA buffer (IBL International). Pyroglutamate-modified Abeta (Abeta_3(pE)–42_) and pan-Abeta42 (Abeta_*x*–42_) were assessed using specific sandwich ELISAs (IBL International).

### 2.7. Aggregated Abeta in Brain

A 10% (w/v) brain homogenate was prepared from each brain sample in 2% (v/v) NP40 in PBS (NaCl 0.138 M, KCl 0.0027 M, pH 7.4) containing 1 mM PMSF and protease inhibitors (Complete Mini, Roche). The 10% brain homogenates were further diluted into buffer to a final concentration that would provide a signal within the linear range of the immunoassay. The concentration of aggregated Abeta was measured using a proprietary assay (Amorfix Aggregated Abeta Assay (A^4^), Amorfix, Mississauga, Canada). Using a proprietary sample enrichment protocol, only the aggregated Abeta was isolated from each sample. Each sample was then disaggregated to allow detection of monomeric Abeta using the Amorfix Abeta immunoassay based on an europium-fluorescent bead coupled to the 4G10 antibody (N-terminal) and magnetic beads coupled to the antibodies 1F8 and 2H12 (C-terminal) recognizing Abeta40 and Abeta42, respectively. The europium fluorescence intensity was measured using Time Resolved Fluorescence (TRF) on each sample in triplicate and is directly proportional to the concentration of Abeta within the sample. The current limit of detection is 50 fg/well. The S/N cutoff value for all experiments was 2.0, equaling two times the background signal from buffer alone.

## 3. Results and Discussion

### 3.1. The APP-Ld Transgenic Mouse Recapitulates Early and Late Hallmarks of Alzheimer's Disease

The APP[V717I] mutation is the most frequent in familial AD with 74 families known, *versus* only three with the APP Swedish mutation (AD&FTD Mutation Database—http://www.molgen.ua.ac.be/ADMutations). In the APP[V717I] mice, the London mutant allele is heterogeneously expressed under control of the neuron-specific murine *thy1 *gene promoter, steering postnatal expression to a level 2 times higher than endogenous APP [9].

### 3.2. Late Stage Amyloid Plaque Pathology, the Object of Historical Focus

#### 3.2.1. Increased Abeta Levels

In the APP-Ld mouse, an age-dependent progressive increase of both soluble and insoluble Abeta40 and Abeta42 levels was observed in brain extracts. Soluble Abeta42/40 ratios increased up to 1 with age, whereas insoluble Abeta42/40 ratios were 5–10 times higher ([11] and unpublished data). Thus, APP-Ld mice display a high relative concentration of Abeta42, the more fibrillogenic Abeta species, essential for amyloid deposition in the parenchyma and vessels [12]. The V717I substitution is located downstream of the gamma-secretase cleavage site and affects the processing of the APP protein causing a shift from Abeta40 to Abeta42 cleavage and thereby increasing the Abeta42/40 ratio. Since processing at the *α*-, *β*-, or *γ*-cleavage sites is not clearly affected by age, the production of Abeta *per se* is not thought to be the primary cause of the accumulation in the APP-Ld mouse brain [11]. Instead, the failure of Abeta clearance or degradation has been proposed as the underlying mechanism.

#### 3.2.2. Parenchymal Amyloid Depositions

**Figure 1:**
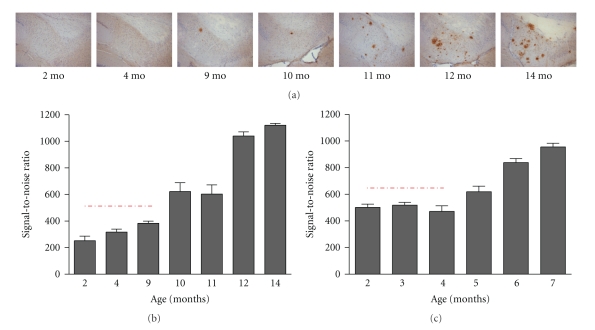
Abeta aggregation prior to plaque formation in APP[V717I] (APP-Ld) and APP[V717I] × PS1[A246E] mice. (a) Representative photo collection of anti-Abeta stained sections showing total plaque load in APP-Ld mice of different ages (proprietary anti-Abeta Nanobody, reMYND/Ablynx, Belgium). (b) Aggregated Abeta in APP-Ld mice of different ages, both in preplaque (indicated by the dashed line) and postplaque stages of the Alzheimer pathology (A^4^-assay, Amorfix Life Sciences Ltd., Mississauga, Canada). The signal of nontransgenic mice was under the S/N cutoff value (data not shown). (c) As in B, for the APP[V717I] × PS1[A246E] model. The signal of nontransgenic mice was under the S/N cutoff value (data not shown).

Parenchymal amyloid plaques arise at the age of 10–12 months in the entorhinal cortex and subiculum (Figure 1(a))—brain regions hierarchically involved in early stages of development of AD pathology in patients [13, 14]—and subsequently spread to the frontal cortex [9]. A proprietary anti-Abeta Nanobody recognizes fibrillar Abeta species with high specificity and affinity. Two types of Abeta depositions—resembling the pathology in AD brain—develop in the brain parenchyma of the APP-Ld mouse, that is, plaques bearing a diffuse character immunoreactive to Abeta antibodies and senile (Thioflavin S positive) plaques comprising an amyloid core surrounded by a halo and neuritic processes [9, 15]. The neuritic component contains hyperphosphorylated forms of protein Tau detected as dystrophic processes by mAb AT8 immunohistochemistry [9] attributed to early neurofibrillary changes in AD. In addition, plaque formation is accompanied by amyloid-associated neuroinflammation, that is, astrocytosis and gliosis, also typically found in AD patients [16], and can be readily detected with immunohistological markers GFAP for staining astrocytes and CD11b/CD45 for the total and activated microglia load, respectively.

#### 3.2.3. Vascular Amyloid Depositions (Cerebral Amyloid Angiopathy, CAA)

At old age, the deposition of amyloid in cerebral vessel walls is observed in the APP-Ld model, with from 10 to more than 50 vessels affected per coronal brain section [17]. Similar to parenchymal plaque formations, Abeta42 is the first peptide to be deposited in vessels, and as such entrapping massive amounts of soluble Abeta40 peptide, the latter ultimately making up for the vast majority of amyloid in vascular plaques [17]. Cerebral amyloid angiopathy is frequent in AD [18] and the ratio of Abeta42/40 is lower in vascular than in parenchymal plaques [19]. The latter is explained by drainage of Abeta40 along the perivascular space because of its higher solubility. The morphological, ultrastructural, and biochemical aspects of the human vascular amyloid depositions, as well as the localization and the type of vessels affected, are reproduced in the APP-Ld model suggesting a similar underlying mechanism of Abeta deposition [17]. Vascular amyloid leads to progressive vessel wall damage and aneurism formation, predisposing the mice to hemorrhage and eventual microbleeds observed at very old age (25–30 months) reminiscent of vascular amyloidosis in a subset of AD patients.

#### 3.2.4. Abeta_3(pE)–42_ Accumulation in Plaques

**Figure 2:**
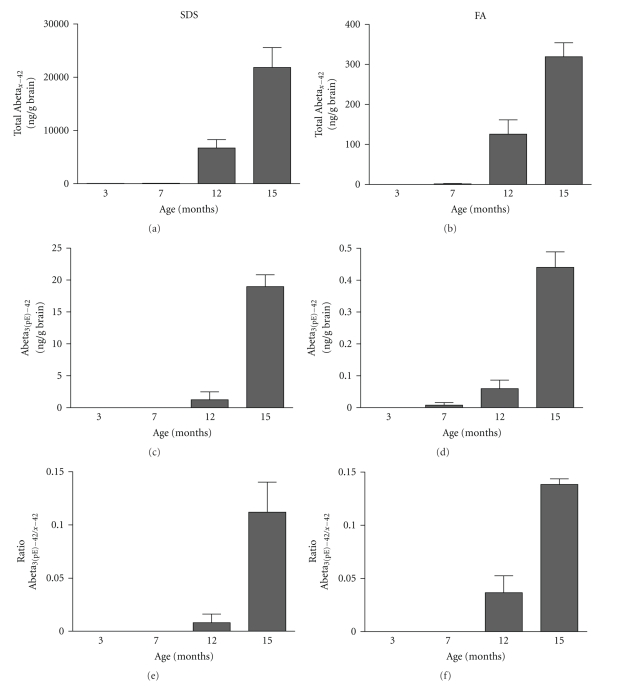
Abeta_3(pE)–42_ determination in the SDS and FA extracts of APP-Ld brain. Pan-Abeta42 (a, b) and Abeta_3(pE)–42_ (c,d) concentrations in SDS (a, c) and FA (b, d), as well as the corresponding Abeta_3(pE)–42_ to pan-Abeta42 ratios (in %) (e, f).

The presence of C-truncated Abeta1-38 and N-truncated Abeta11–42 peptides in APP-Ld brain extracts was demonstrated previously in [20]. In addition, we show here that pyroglutamate-modified Abeta3–42 (Abeta_3(pE)–42_), a dominant fraction of Abeta peptides in senile plaques of AD brain [21], is detected in the insoluble fraction of brain extracts of APP-Ld mice from the age of 12 months onwards (Figure 2). Although absolute levels are low in both extracts, an age-dependent increase was observed. These N-terminal truncated Abeta species are abundant in amyloid deposits in sporadic and familial AD [22]. They resist proteolysis, accelerate aggregation by seeding and thereby entrapping other Abeta forms, and are claimed to be neurotoxic [23]. Reduction of Abeta_3(pE)–42_ by inhibition of glutaminyl cyclase (QC), the enzyme catalyzing the N-terminal pGlu modification, was therefore suggested as a new therapeutic target in AD [24].

#### 3.2.5. Decreased CSF Abeta42/40 Ratio with Age

**Figure 3:**
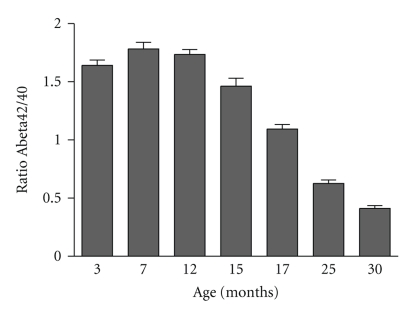
Progression of CSF Abeta42/40 ratios in ageing APP-Ld mice; from the age of 15 months onwards, the ratio of Abeta42/40 in CSF decreases.

In parallel with what is observed in AD patients [25–27], the ratio of Abeta42/40 in CSF decreases in ageing APP-Ld mice (Figure 3), correlating in time with the appearance of abundant parenchymal and vascular plaque formation from the age of 15 months onwards. The ratio Abeta42/40 in CSF could be a more valuable diagnostic tool for early-stage AD and mild cognitive impairment (MCI) patients [25].

### 3.3. Early Stage Preplaque Pathology, the Object of More Recent Focus

#### 3.3.1. Cognitive and Behavioral Impairment

From the age of three months onwards, robust and significant spatial and nonspatial orientation and memory deficits are observed in the APP-Ld model, which does not relate to the timing of plaque deposition [9]. Accordingly, impaired NMDA-dependent long-term potentiation and decreased NMDA-receptor activation in hippocampal CA1 region have been demonstrated at a preplaque age stadium. Inhibiting APP processing rescued this effect suggesting the dysfunction of the glutamate neurotransmitter system represents a pathologically relevant process secondary to Abeta toxicity involving synaptic plasticity and memory formation [9, 28, 29]. Also derangement of associative learning, hyperactivity, anxiety, and aggression develops independently of plaque formation [30]. Some of these have been shown to be alleviated by serotonergic drugs [31]. Premature death caused by epileptic seizures is an epiphenomenon observed in Alzheimer patients [32, 33] and is also found in the APP-Ld model [9].

The preplaque synaptic and cognitive demise and related behavioral deficits in the APP-Ld mouse dissociate the late fibrillar amyloid plaques from pathobiological processes at early age apparently elicited by specific soluble Abeta forms inducing neuronal toxicity (see also “Abeta aggregation prior to plaque formation”).

A similar dissociation of amyloid plaque pathology and behavioral aspects was observed in mild cognitive impairment (MCI) patients [34]. In addition, occurrence of soluble aggregated Abeta forms—rather than the amyloid plaques—in AD brain correlates in time with the onset of cognitive decline in AD patients [35].

#### 3.3.2. Brain Inflammation

In AD patients, neuroinflammation is recognized as an early defect in the pathogenesis [36]. In the APP-Ld model, early inflammation is evident as foci of activated microglia and astroglia randomly distributed throughout hippocampus and cortex, from the age of 3 months [37]. These foci have been proposed to represent the earliest sites of amyloid deposition, likely evolving into amyloid plaques. Moreover, these early and focal inflammatory events have been postulated to contribute to neuronal dysfunction at a young age and to the early cognitive impairment in the APP-Ld model. Interestingly, neuronal BACE1 expression was demonstrated to be upregulated in close proximity of activated microglia and astrocytes, strongly pointing to an interaction between neurodegenerative and neuroinflammatory events [37].

#### 3.3.3. Abeta Aggregation Prior to Plaque Formation

Already at the age of two months, that is, long before the onset of amyloid deposition and plaque formation at 10–12 months, soluble oligomeric Abeta is present in brain of APP-Ld mice and their levels gradually increase with age (Figure 1(b)). Determination of the exact nature of these aggregated Abeta species in the brain of APP-Ld mice, and their correlation with the significant cognitive deficits, is the subject of current investigations.

The coincidental timing with the onset of cognitive defects is intriguing, given that in diseased brains increases of soluble Abeta also correlate with cognitive decline and neuropathological hallmarks of AD [35]. A causal link of Abeta oligomers has also been suggested by a study where clearing of plaques, but not oligomers, did not mitigate cognitive decline [38]. The realization of Abeta oligomers representing noxious species has spurred research to find ways to inhibit their formation as a strategy for therapeutic intervention.

#### 3.3.4. Tau Phosphorylation

In later stages of the pathology in APP-Ld mice, plaque-associated dystrophic neurites develop containing hyperphosphorylated murine Tau [9]. Recent data have demonstrated GSK3*β*-activation and mouse Tau phosphorylation in the hippocampus of the single APP-Ld model at a preplaque stadium ([1, 39] and unpublished data). These findings correlate with the presence of soluble Abeta aggregates and cognitive deficits suggesting an “instigator role” of Abeta in the downstream pathobiology mediated by kinases resulting in Tau phosphorylation. Although the exact nature of the responsible Abeta form(s) remain(s) elusive, an unspecified subset of soluble oligomeric species is hypothesized to be involved in the neurotoxicity cascade [38]. Relevant in this context, knockout of the murine Tau prevented decline of learning and memory as well as hypersensitivity to excitotoxicity in a independent APP transgenic mouse model [40]. The combined data support the hypothesis that endogenous Tau is required for Abeta-mediated neurotoxicity in mice, illustrating a pathobiological interplay between Abeta and Tau in transgenic APP mice.

### 3.4. The *A* *P* *P*[*V*717*I*] × *P* *S*1[*A*246*E*] Model for Faster Ranking of Lead Molecules

Complementary to the late onset APP-Ld single transgenics, an APP[V717I] × PS1[A246E] bigenic model (APP*PS1) has been developed being a more aggressive model with accelerated amyloid pathology [11]. These mice carry additionally the human PS1[A246E] transgene, also under control of the murine *thy1 *gene promoter, containing a clinical mutation in the region encoding the transmembrane (AD&FTD Mutation Database—http://www.molgen.ua.ac.be/ADMutations). 

Whereas in ageing APP-Ld mice both Abeta42 and Abeta40 are increased, in the double transgenic APP[V717I] × PS1[A246E] mice the more hydrophobic Abeta42 is preponderantly increased. Consequently, accelerated amyloid plaque pathology is observed, caused by the higher Abeta42/40 ratio (above 1), with amyloid plaque development at the age of 5–6 months [41].

Concomitant to the increased Abeta42 production in the APP*PS1 double transgenic model, soluble aggregated forms of Abeta during the preplaque stadium were found two-fold increased at the age of 2–4 months compared to the single APP-Ld model (Figure 1(c)). The aggravated Abeta pathobiology in the APP*PS1 double transgenic mouse recapitulates the effect of the early-onset familial Alzheimer disease (EOFAD) PS1 mutations on the metabolism of APP [42].

Practical advantage of the double APP*PS1 *versus* the single APP-Ld model involves the shorter time span between onset of amyloidosis and amyloid plaque development and thereby the relatively faster evaluation and prioritization of lead molecules before further drug development.

### 3.5. Combined and Age-Dependent Development of Amyloid Plaques and Neurofibrillary Tangles in *A* *P* *P*[*V*717*I*] × *T* *A* *U*[*P*301*L*] Double Transgenic Mice

Tau mutations are linked with FTD in humans, evoking a tauopathy reminiscent of that in AD, including cognitive defects and neurodegeneration leading to dementia. In total, 32 families have been identified carrying this point mutation in exon 7 (AD&FTD Mutation Database—http://www.molgen.ua.ac.be/ADMutations). 

In the single TAU[P301L] transgenics, the observed conformational change and age-dependent accumulation of AT8 and AT100 reactive insoluble Tau have been proposed to trigger an age-dependent tangle pathology (starting at age 8–9 months) [10, 43]. In parallel the TAU[P301L] mice develop motor deficits like limb clasping and impaired survival, that is, succumbing before age 12–13 months [10]. Although this model does not contain any amyloid-related hallmarks, it has been proven very suitable for therapeutic testing of candidate drugs aimed at Tau pathology ([10] and unpublished data).

To introduce Tau pathology in the APP-Ld mouse, the bigenic APP[V717I] × TAU[P301L] mouse line (APP*TAU) was created [1, 10]. Three important characteristics of AD are recapitulated in this model: intracellular neurofibrillary tangles (NFTs), extracellular amyloid plaques, and cognitive impairments. The amyloid pathology in these mice is more intense than in the single APP-Ld mouse, but similar in its aspects and timing [1]. NFT pathology is significantly enhanced in the hippocampus and cortex relative to the parental single TAU[P301L] model, developing in the same time frame as the amyloid plaques [1].

The bigenic APP*TAU model will be highly valuable for further investigating the molecular interplay between Abeta and protein Tau in causing neurodegeneration and as a tool to evaluate drug candidates.

## 4. Conclusions

During the past decades, a variety of rodent models have been developed and proven to be valuable tools for deciphering the complexity of AD and contributed to the discovery and development of diagnostic and therapeutic innovations. However, the current AD animal models have their limitations and may, at least partly, contribute to the high failure rates of AD drug candidates in clinical trials. Apart from the obvious gap between mice and men, the translational gap also stems from differences in etiological factors, spatio-temporal onset of pathology, and brain physiology [6]. 

Notwithstanding the above, the AD mouse models are of utmost importance for the exploration of novel therapeutic approaches. Given the particularities and limitations of the animal models, the single most important parameter for meaningful proof-of-concept studies is the selection of the most appropriate animal model which most faithfully recapitulates the key parameters of disease. In addition, preclinical proof-of-concept studies should ideally be performed in more than one model to capture as much disease pathology as possible and to discern animal and model specific artifacts. For instance, by analyzing two models with different APP mutations, compounds acting specific in the context of one particular mutation would be distinguished.

The APP-Ld mouse model for amyloid pathology represents a highly valuable model for drug testing, especially when targeting the amyloid cascade, but also for modulators of beginning Tau pathology. As summarized in this paper, the processing of human APP-Ld and production of Abeta in APP-Ld mice result in a plethora of pathological and behavioral effects modeling key disease parameters of AD.

Development of amyloyd plaque pathology in brain parenchyma and vasculature, and related inflammatory processes (astrocytosis, microgliosis) arise in an age-dependent way. Concomitant clearance of Abeta to CSF is affected as a presumed consequence of massive deposition of Abeta. Thus, the resultant decrease of Abeta CSF (in function of age) closely mimics the situation in AD patients and provides an efficacy biomarker in preclinical studies directed to evaluate Abeta-modulating drugs. 

The novel data on the presence of insoluble pyroglutamate-modified Abeta3–42 in the brain of aged APP-Ld mice offer alternative therapeutic options. N-terminal truncated Abeta is highly abundant in AD brain and is believed to be an initiator of the Abeta aggregation cascade because of its exceptional physical properties. The conservation of the QC mediated posttranslational modification process provides unique opportunities to study the role of pyroglutamate-modified Abeta in AD and for testing novel QC inhibitors for therapeutic potential.

Early formed soluble aggregates of Abeta (as of 2 months) in brain of APP-Ld mice suggest that these Abeta misconformers are primary triggers of synaptic and neurotoxicity. The similarity with AD patients is striking, especially in light of recent findings demonstrating that soluble oligomers species were elevated in AD brain and appear to correlate with cognitive decline and neuropathological hallmarks [35]. Although the exact nature of the toxic Abeta species remains elusive, more than 25 years after their discovery, this finding can have an important impact on drug development strategies aimed at Abeta pathobiology. 

Abeta-mediated activation of GSK3*β*-kinases and phosphorylation of endogenous mouse Tau in APP-Ld mouse brain reflect an intriguing and potentially very important connection between Abeta and Tau pathology [1]. Thus, the APP-Ld transgenics also model disease relevant Tau pathology [40] and would permit studies of Abeta effects on Tau pathology and assessing the therapeutic potential of Tau modulators.

Collectively, APP-Ld mice recapitulate the AD-related development and progression of Abeta pathobiology and its downstream effects on cognition and Tau most closely, however, without neurofibrillary tangles and massive neuronal loss. This positions the APP-Ld mouse model as a valuable tool for detecting and analyzing Abeta and Tau modulating AD drugs with the potential to fundamentally modify the course of the disease. By combining APP-Ld with mutant PS1, a further aggravated Abeta pathology is obtained providing practical advances for especially proof-of-concept studies of drug candidates. The APP-Ld*TAU-P301L double transgenics offer the advantage of a more complete pathology facilitating research and drug development focusing or addressing the interplay of Abeta and Tau in onset and progression of AD.
